# Kidney Function, Kidney Replacement Therapy, and Mortality in Men and Women

**DOI:** 10.1016/j.ekir.2021.12.024

**Published:** 2021-12-27

**Authors:** Sebastian Hödlmoser, Juan Jesus Carrero, Amelie Kurnikowski, Edouard L. Fu, Oskar Swartling, Wolfgang C. Winkelmayer, Eva S. Schernhammer, Manfred Hecking

**Affiliations:** 1 Department of Epidemiology, Center for Public Health, Medical University of Vienna, Vienna, Austria; 2 Clinical Division of Nephrology & Dialysis, Department of Internal Medicine III, Medical University of Vienna, Vienna, Austria; 3 Department of Medical Epidemiology and Biostatistics, Karolinska Institutet, Stockholm, Sweden; 4 Department of Clinical Epidemiology, Leiden University Medical Center, Leiden, The Netherlands; 5 Clinical Epidemiology Division, Department of Medicine, Karolinska Institutet, Stockholm, Sweden; 6 Section of Nephrology, Selzman Institute for Kidney Health, Baylor College of Medicine, Houston, Texas, USA; 7 Department of Epidemiology, Harvard T.H. Chan School of Public Health, Boston, Massachusetts, USA; 8 Channing Division of Network Medicine, Brigham and Women’s Hospital and Harvard Medical School, Massachusetts, USA

**Keywords:** kidney replacement therapy initiation, mortality in the predialysis stage, sex and gender disparity, sex/gender differences in nephrology

## Abstract

**Introduction:**

Women are more likely to have chronic kidney disease (CKD), compared with men, yet they are less likely to receive dialysis. Whether this sex disparity, which has predominantly been observed in nephrology-referred or CKD-specific cohorts so far, has a biological root cause remains unclear.

**Methods:**

We extracted general population data from the Stockholm CREAtinine Measurements project (SCREAM) (*N =* 496,097 participants, 45.5% men, 54.5% women). We used Cox regression to model male-to-female cause-specific hazard ratios (csHRs) for the competing events kidney replacement therapy (KRT, by dialysis or transplantation) and pre-KRT death, adjusted for baseline age, baseline kidney function (assessed via estimated glomerular filtration rate [eGFR] and eGFR slope), and comorbidities. Furthermore, we modeled sex-specific all-cause mortality by eGFR, again adjusted for age, eGFR slope, and comorbidities at baseline.

**Results:**

Compared with women, men were significantly more likely to receive KRT (fully adjusted male-to-female csHR for KRT 1.41 [95% CI 1.13–1.76]) but also more likely to experience pre-KRT death (csHR 1.36 [95% CI 1.33–1.38]). Differences between men and women regarding all-cause mortality by eGFR indicated a higher mortality in men at low eGFR values.

**Conclusion:**

Our data show that sex differences in CKD outcomes persist even after controlling for important comorbidities and kidney function at baseline. While future studies with a wider range of biological factors are warranted, these data suggest that nonbiological factors may be more important in explaining existing sex disparities in CKD progression and therapy.

## Graphical abstract

**Figure:**
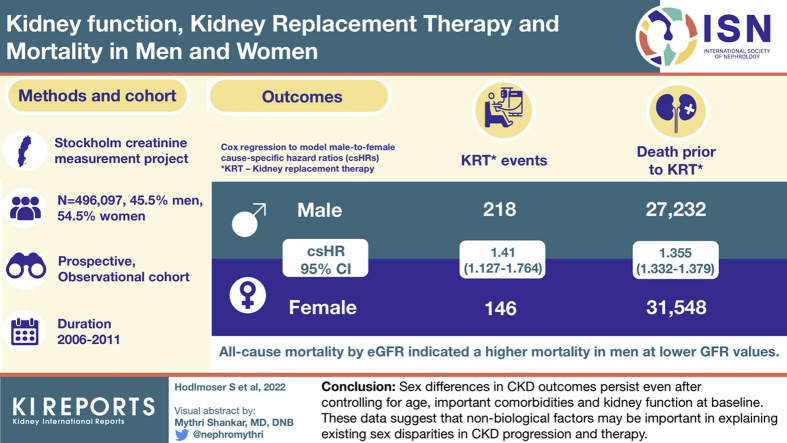

CKD is one of the fastest growing public health concerns in recent history, in consequence of aging populations and increasing burdens of CKD risk factors such as obesity, hypertension, and diabetes.^1^^,^^2^ In a 2016 review and meta-analysis, the global prevalence of CKD stage G3 to G5 was estimated to be 10.6%, and, importantly, consisted of a prevalence of 8.1% for men but 12.1% for women.^3^ The importance of addressing sex and gender differences^4^ in medicine^5^, ^6^, ^7^ is now increasingly recognized in nephrology.^8^, ^9^, ^10^ For more than 2 decades, it has been noted that while there are more women than men with CKD, there are fewer women than men among those starting KRT through dialysis or transplantation.^8^^,^^11^, ^12^, ^13^, ^14^

The discrepancy between a higher prevalence of CKD in women compared with men and a lower incidence of KRT initiation for women is not well understood. Preliminary explanations for this sex discrepancy are related, on the one hand, to biological sex differences in CKD progression rates,^15^^,^^16^ or, on the other hand, to sex differences in the management of comorbidities or quality of care.^4^^,^^17^ Biological variables that have been hypothesized to differ between men and women include different patterns of morbidity^8^ and GFR, which is the most commonly used measure of CKD severity. Progression of CKD is usually expressed as change in eGFR over time, and this has been shown to occur more rapidly in men than in women.^15^ While the relationship between eGFR and mortality has been previously examined in a sex-specific manner,^18^ the sex-specific criteria for receiving KRT are less well studied.

A possible alternative explanation for the existing sex differences in CKD outcomes is that women are more likely to opt for conservative care^8^^,^^17^ and might be more likely to die than to start dialysis compared with men (i.e., death as a competing risk to KRT initiation). To date, research on these issues has occurred primarily in cohorts of patients referred to nephrology care, a health care process with well-described sex differences.^8^ However, the underlying causes may be rooted at the broader population level, and adverse events in women may have occurred primarily in the larger segment of the population with CKD that has never seen a nephrologist.^15^

Against this background, we analyzed a population-based cohort representative of the metropolitan Stockholm region to examine the risks of death or KRT among men and women. We explored the role of a variety of measured biological factors in mediating possible differences between the sexes and whether death as competing risk for KRT initiation differed between men and women.

## Methods

### Study Population

The SCREAM is a population-based, prospective cohort study of residents of Stockholm, conducted from 2006 to 2011. In the SCREAM data set, all individuals who accessed health care and underwent creatinine assessments in connection to a health care encounter are included. Through the unique personal number of each citizen, this repository was linked to the regional health care database (allowing to withdraw complete information on comorbidities with International statistical Classification of Diseases and related health problems, version 10 diagnoses and health care utilization until end of 2012), the Swedish population registry (allowing to monitor death risk, available to us until the end of 2012), and the Swedish renal registry (allowing to ascertain initiation of KRT until the end of 2012). The representativeness of SCREAM and its protocol have previously been described.^19^ The study utilized only deidentified data and thus was deemed not to require informed consent, being approved by the regional ethical review boards and the Swedish National Board of Welfare.

### Study Cohort

For this analysis, we included only residents with known age and sex, who underwent creatinine testing in primary or secondary care and were not on dialysis or had a history of kidney transplantation at baseline. We did not consider inpatient creatinine measurements, as they may be influenced by disease severity and might not represent stable kidney function. Furthermore, we only included records where subjects were above the age of 45 years at the time of creatinine measurement because routine creatinine testing, KRT, and death before this age are uncommon.

### Main Exposure and Kidney Function Covariates

The exposure of interest was sex as registered in the participants’ personal identification number, which was identified by the sequence of registry numbers. We note that registered sex may be changed throughout life if the citizen wishes to be recognized otherwise.^20^ We also note that the binary variable recorded in SCREAM does not differentiate between sex (male vs. female) and gender (man vs. woman) or transgender.^21^ Throughout the current manuscript, SCREAM participants of male and female sex (assuming that this distinction will be accurate for most individuals in the absence of genetic testing) are referred to as men and women, respectively, to remain consistent with previous work.^11^^,^^22^

To assess kidney function for each participant at baseline, we used eGFR (ml/min per 1.73 m^2^) and eGFR slope (i.e., change in eGFR in ml/min per 1.73 m^2^ per year). On the basis of outpatient creatinine (in μmol/l) tests in Stockholm primary or secondary care, we calculated eGFR using the CKD-Epidemiology Collaboration formula from the year 2009.^23^ Collecting data on ethnicity is not allowed in Swedish health registries, which is why we did not correct the equation for race. For an estimate of eGFR slope, we used the first year of eGFR observations of each individual to fit a linear mixed effects regression model for eGFR, with intercept and time as independent variables and random intercept and slope per patient. We defined eGFR slope as the combination of the fixed and random effect of the slope parameter per subject. We then set the study baseline for our analyses to the first outpatient creatinine record after at least 1 year of observation (hence, the first eGFR measurement per patient that was not used for the slope estimation). Thus, at baseline for the subsequent time-to-event analyses, we had both an assessment of the participant’s kidney function (in form of eGFR) and previous slope of eGFR decline. We note that this strategy implied that participants had to undertake at least 2 eGFR assessments and had to be observed for at least 1 year in the database.

### Additional Covariates

Further covariates calculated at baseline included age and the presence of comorbidities. We derived dichotomous comorbidity variables (yes/no) from International statistical Classification of Diseases and related health problems, version 10 codes using the comorbidity domains detailed in the Elixhauser score^24^ (e.g., cardiovascular diseases [CVDs], diabetes, hypertension, cancer types; see Table 1 for full list, Supplementary Table S1 for International statistical Classification of Diseases and related health problems, version 10 codes used). We defined CVD as the composite of congestive heart failure, peripheral vascular disorders, or valvular disease. When defining these chronic comorbidities, we imposed no time limit and evaluated all issued diagnoses since the implementation of the International statistical Classification of Diseases and related health problems, version 10 in Sweden in 1997.

**Table 1:** Study population characteristics

Parameter	Men	Women	All
	*n =* 225,971	*n =* 270,126	*N =* 496,097
Age	63.9 [16.4]	65.5 [19.7]	64.7 [18.2]
Creatinine [μmol/l]	82.0 [21.0]	66.0 [18.0]	73.0 [23.0]
eGFR [ml/min per 1.73 m^2^]	86.4 [22.8]	83.4 [25.2]	84.8 [24.2]
CKD stage, *n* (%)			
G0–G2	200,875 (88.9)	230,999 (85.5)	431,874 (87.1)
G3	22,691 (10)	36,030 (13.3)	58,721 (11.8)
G4	2012 (0.9)	2733 (1)	4745 (1)
G5	393 (0.2)	364 (0.1)	757 (0.2)
eGFR slope [ml/min per 1.73 m^2^/yr]	−1.6 [0.9]	−1.7 [0.9]	−1.7 [0.9]
Cardiovascular disease, *n* (%)	15,003 (6.6)	13,087 (4.8)	28,090 (5.7)
Congestive heart failure, *n* (%)	1034 (0.5)	946 (0.4)	1980 (0.4)
Peripheral vascular disorders, *n* (%)	9929 (4.4)	7998 (3)	17,927 (3.6)
Valvular disease, *n* (%)	5681 (2.5)	5218 (1.9)	10,899 (2.2)
Cardiac arrhythmias, *n* (%)	31,038 (13.7)	30,564 (11.3)	61,602 (12.4)
Hypertension, *n* (%)	87,154 (38.6)	108,757 (40.3)	195,911 (39.5)
Pulmonary circulation disorders, *n* (%)	2821 (1.2)	3292 (1.2)	6113 (1.2)
Paralysis, *n* (%)	1560 (0.7)	1255 (0.5)	2815 (0.6)
Other neurologic disorders, *n* (%)	8099 (3.6)	8101 (3)	16,200 (3.3)
Chronic pulmonary disease, *n* (%)	16,518 (7.3)	25,079 (9.3)	41,597 (8.4)
Diabetes, *n* (%)	33,630 (14.9)	26,583 (9.8)	60,213 (12.1)
Hypothyroidism, *n* (%)	4998 (2.2)	30,296 (11.2)	35,294 (7.1)
Liver disease, *n* (%)	5881 (2.6)	5102 (1.9)	10,983 (2.2)
Peptic ulcer disease, *n* (%)	2568 (1.1)	2558 (0.9)	5126 (1)
AIDS/HIV, *n* (%)	426 (0.2)	138 (0.1)	564 (0.1)
Lymphoma, *n* (%)	2184 (1)	2160 (0.8)	4344 (0.9)
Metastatic cancer, *n* (%)	3131 (1.4)	6183 (2.3)	9314 (1.9)
Solid tumor without metastasis, *n* (%)	25,296 (11.2)	31,068 (11.5)	56,364 (11.4)
Rheumatoid arthritis/collagen vascular disease, *n* (%)	6550 (2.9)	15,367 (5.7)	21,917 (4.4)
Coagulopathy, *n* (%)	3819 (1.7)	3288 (1.2)	7107 (1.4)
Obesity, *n* (%)	3197 (1.4)	5491 (2)	8688 (1.8)
Weight loss, *n* (%)	970 (0.4)	1385 (0.5)	2355 (0.5)
Fluid and electrolyte disorders, *n* (%)	3100 (1.4)	6028 (2.2)	9128 (1.8)
Blood loss anemia, *n* (%)	1064 (0.5)	2374 (0.9)	3438 (0.7)
Deficiency anemia, *n* (%)	3142 (1.4)	6039 (2.2)	9181 (1.9)
Alcohol abuse, *n* (%)	12,163 (5.4)	6259 (2.3)	18,422 (3.7)
Drug abuse, *n* (%)	2467 (1.1)	2180 (0.8)	4647 (0.9)
Psychoses, *n* (%)	2670 (1.2)	3716 (1.4)	6386 (1.3)
Depression, *n* (%)	16,207 (7.2)	34,327 (12.7)	50,534 (10.2)

CKD, chronic kidney disease; eGFR, estimated glomerular filtration rate; IQR, interquartile range.

Study population characteristics at study baseline, by sex and overall; median (IQR) for continuous; absolute and relative frequencies (%) for categorical variables.

### Outcomes

The primary outcomes were time from baseline to the competing events KRT (i.e., dialysis initiation or kidney transplant) or death, whichever occurred first, and the respective other outcome was considered a censoring event. The secondary outcomes of interest were time from baseline to death, of any cause and without considering any competing event. All time-to-event data were censored after a maximum of 6 years of follow-up or at study end, that is, when the data collection of the death records and the KRT events stopped (December 2012).

### Statistical Analysis

We summarized study population characteristics overall and by sex using means and SDs for continuous variables and counts and frequencies for categorical data. For the competing events KRT and pre-KRT death, we reported overall counts, unadjusted event rates per 100,000 person years, and age-standardized event rates (to the Stockholm population of 2009^25^) as well as the absolute count of all-cause death events, by sex.

For the primary outcome, we calculated male-to-female csHRs for the competing events of KRT initiation and death before KRT using Cox proportional hazard models. To examine whether “biological” factors influence the chances of KRT and risks of pre-KRT death between sexes, we fitted incrementally adjusted models, from unadjusted (which only used sex as exposure) to “fully” adjusted (further adjusting for age at baseline, all available comorbidities, eGFR at baseline and eGFR slope at baseline). Continuous variables (eGFR, eGFR slope, age) were incorporated via restricted cubic spline terms (the number of knots and knot placement was calculated by the rcs function of the rms library). To visualize differences in death and KRT initiation between men and women over time, we fitted a Fine and Gray model with sex as the only covariate and plotted the cumulative incidence functions for the 2 events separately for each sex. To assess possible effect modification on the multiplicative scale, we estimated male-to-female csHRs with respect to subgroups of age at baseline <60 years (yes/no), CVD (yes/no), diabetes (yes/no), and baseline eGFR <60 ml/min per 1.73 m^2^ (yes/no).

To investigate whether mortality with respect to kidney function differed between men and women, we further fitted Cox proportional hazard models for all-cause death by baseline eGFR, with an eGFR reference level of 95 ml/min per 1.73 m^2^. We adjusted the models for age at baseline, prior eGFR slope, the interaction term sex × eGFR, and comorbidities. Again, continuous variables (eGFR, eGFR slope, age) were included via restricted cubic spline terms. We fitted a “full” model, which included all subjects as well as separate within-sex models for men and women. Similar to the primary analysis, we repeated the all-cause death survival analysis but incorporated interaction terms for diabetes and sex, as well as CVD and sex (in separate models), and compared the hazard ratios by eGFR between those subgroups.

Multiplicative interactions were tested using single parameter or joint Wald tests. Two-sided *P* < 0.05 were considered to be significant. All statistical analyses were conducted in R 4.0.2.^26^

### Sensitivity Analyses

For the primary analysis, we conducted a series of sensitivity analyses. First, instead of the 45-year cutoff for medical records to be included in the study cohort, we used both 40 and 50 years as a cutoff. Second, we excluded eGFR slope from our models and included subjects where eGFR slope estimation was not feasible (i.e., subjects observed only once, or for <1 year). Third, we changed the timescale of the Cox models to the subject’s age (via Cox models with flexible entrance times per patient). Finally, we separated the KRT events into transplantation and dialysis initiation and treated them as distinct competing events.

## Results

**Figure 1:**
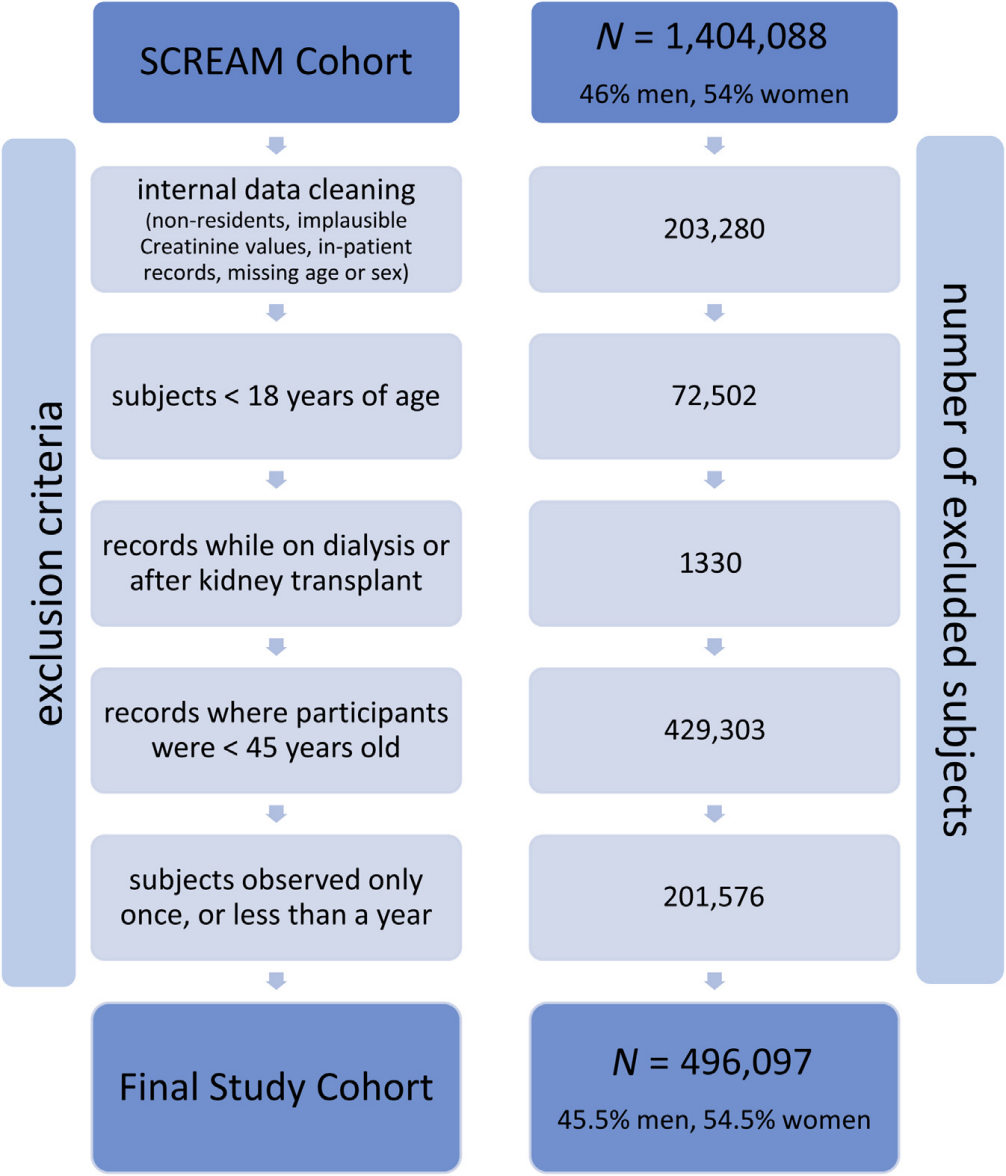
Study flowchart. SCREAM, Stockholm CREAtinine Measurements Project.

Baseline characteristics of the 496,097 subjects fulfilling the inclusion criteria are shown in Table 1, overall and by sex. The study sample consisted of 45.5% men and 54.5% women (see Figure 1 for detailed study flowchart). On average, women were older than men and had lower creatinine and higher eGFR values. While women were classified as having CKD stages G3 to G4 more often than men, both the relative and absolute number of subjects with CKD stage G5 was higher in men. The estimated eGFR slope was similar between sexes. Regarding comorbidities, diabetes (men vs. women: 14.9% vs. 9.8%), liver disease (2.6% vs. 1.9%), alcohol abuse (5.4% vs. 2.3%), and AIDS/HIV (0.2% vs. 0.1%) were more prevalent among men, while hypothyroidism (2.2% vs. 11.2%), rheumatoid arthritis (2.9% vs. 5.7%), fluid disorders (1.4% vs. 2.2%), blood loss anemia (0.5% vs. 0.9%), and depression (7.2% vs. 12.7%) were more prevalent among women. The total observation period amounted to 828,749 person years for men and 997,275 person years for women. Median follow-up was similar for both sexes (men: 3.90 years [interquartile range 2.59–4.86], women: 3.92 years [interquartile range 2.64–4.87]). Unadjusted event rates per 100,000 person years for men and women amounted to 26.3 and 14.6 for KRT, and 3285.9 versus 3163.4 for death before KRT, respectively. Age-standardized event rates were very similar to unadjusted event rates for KRT (men: 25.9, women: 14.6), whereas age-standardized pre-KRT death events were higher in women (men: 2668.9, women: 2740.1) (Table 2).

**Table 2:** Crude and standardized even rates

Parameter	Men	Women
PY	828,749	997,275
Median follow-up (IQR)	3.90 (2.59–4.86)	3.92 (2.64–4.87)
KRT events	218	146
Per 100,000 PY	26.3	14.6
Age-standardized	25.9	14.5
Pre-KRT death events	27,232	31,548
Per 100,000 PY	3285.9	3163.4
Age-standardized	2668.9	2740.1
All-cause deaths events	27,300	31,604

IQR, interquartile range; KRT, kidney replacement therapy; PY, person year.

PY, median follow-up (first and third quartile), event counts, unadjusted event rates per 100,000 PY and event rates age-standardized to Stockholm Population in 2009, of KRT and pre-KRT death, and all-cause death events, per sex.

### Primary Outcome: Competing Risks of KRT and Death

**Figure 2:**
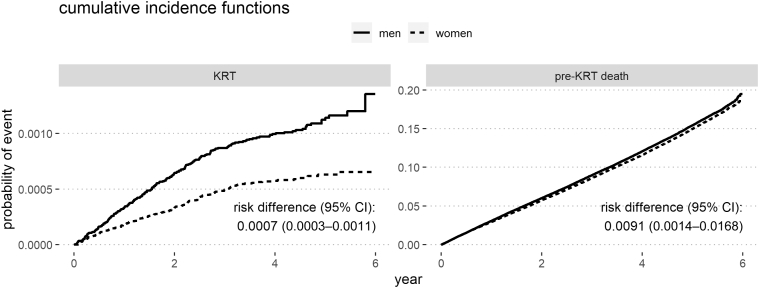
Fine and Gray cumulative incidence functions of KRT and pre-KRT death, per sex; risk difference with 95% CI at 6 years. KRT, kidney replacement therapy.

The csHRs with 95% CIs of the competing events KRT and pre-KRT death as well as all-cause death hazard ratios with 95% CIs are shown in Table 3, for incrementally adjusted models. The unadjusted male-to-female csHR for KRT was 1.80 (95% CI 1.46–2.21, model 1); after full adjustment, this csHR decreased to 1.41 (95% CI 1.13–1.76, model 7). The unadjusted male-to-female HR for pre-KRT death was 1.04 (95% CI 1.02–1.06, model 1) but increased to 1.36 (95% CI 1.33–1.38, model 7) when fully adjusted, with age adjustment exerting the most prominent effect. The all-cause death hazard ratios were very close to the pre-KRT csHRs. Figure 2 visualizes the cumulative incidences of the competing events by sex and the absolute risk differences at 6 years with 95% CIs.

**Table 3:** Cause-specific hazard ratios of competing events

Parameter	Adjustments	KRT m-to-f csHR	Pre-KRT death m-to-f csHR	All-cause death m-to-f HR
Model 1	Unadjusted	1.80 (1.46–2.21)	1.04 (1.02–1.06)	1.04 (1.02–1.06)
Model 2	Age	1.74 (1.41–2.15)	1.46 (1.44–1.49)	1.46 (1.44–1.49)
Model 3	Age + diabetes	1.56 (1.26–1.92)	1.44 (1.41–1.46)	1.44 (1.41–1.46)
Model 4	Age + diabetes + hypertension	1.58 (1.28–1.96)	1.43 (1.41–1.46)	1.43 (1.41–1.46)
Model 5	Age + diabetes + hypertension + CVD	1.54 (1.25–1.91)	1.40 (1.37–1.42)	1.40 (1.37–1.42)
Model 6	Age + diabetes + hypertension + CVD + eGFR + eGFR slope	1.38 (1.11–1.71)	1.39 (1.37–1.41)	1.39 (1.37–1.41)
Model 7	Age + diabetes + hypertension + CVD + eGFR + eGFR slope + 23 comorbidities	1.41 (1.13–1.76)	1.36 (1.33–1.38)	1.35 (1.33–1.38)

csHR, cause-specific hazard ratio; CVD, cardiovascular disease; eGFR, estimated glomerular filtration rate; f, female; HR, hazard ratio; KRT, kidney replacement therapy; m, male.

m-to-f csHR with 95% CIs of the competing events KRT and pre-KRT death, as well as all-cause death HRs with 95% CIs; CVD denotes cardiovascular disease as congestive heart failure, peripheral vascular disorders, or valvular disease; model 7 includes diabetes, hypertension, the 3 CVD factors, and all other 23 comorbidities depicted in Table 1.

### Subgroup Analyses

**Figure 3:**
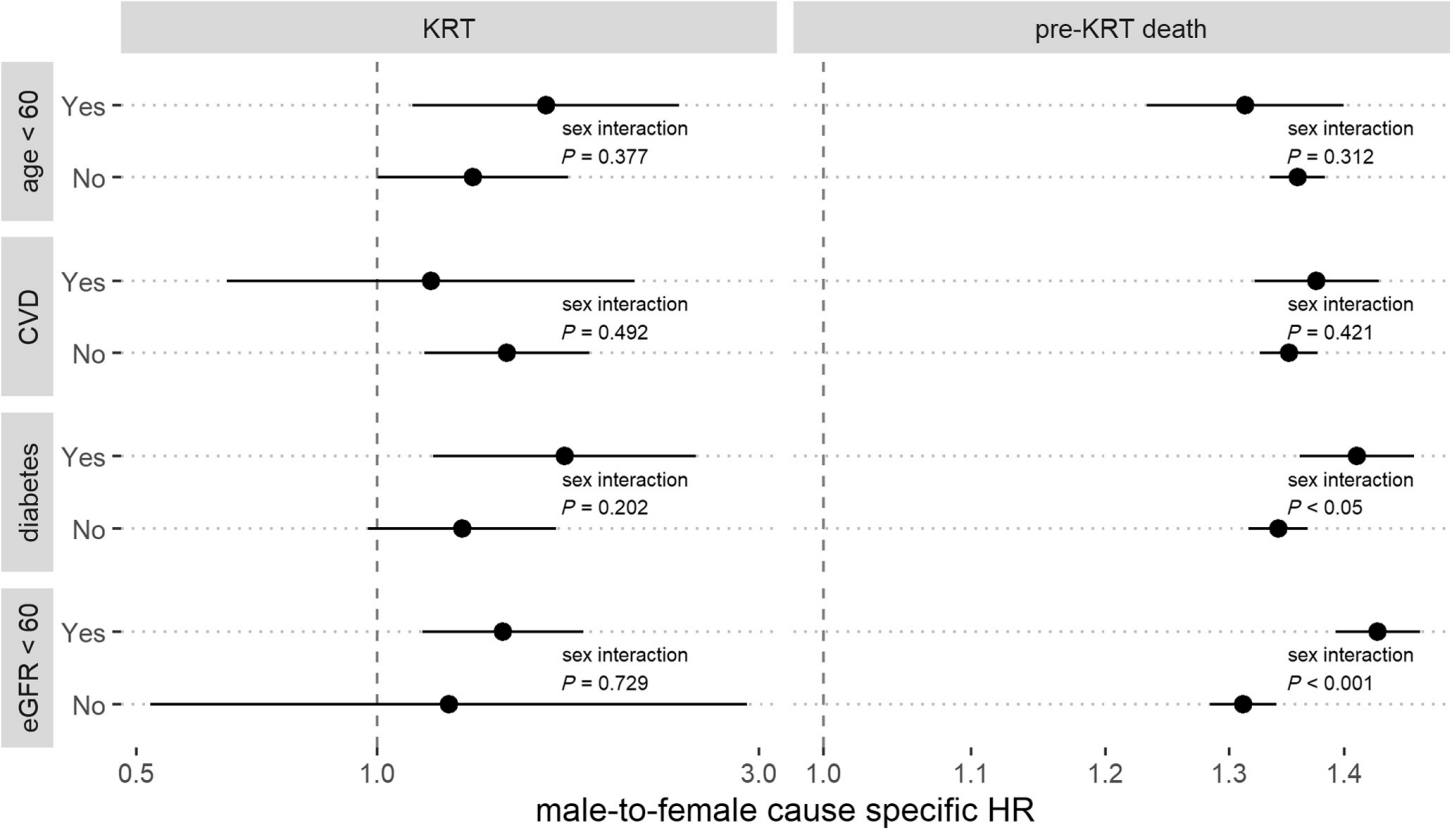
Male-to-female cause-specific HRs and 95% CIs of the competing events KRT and death before KRT between subjects above or below 60 years of age at baseline, with/without CVD (i.e., congestive heart failure, peripheral vascular disorders or valvular disease), diabetes, and baseline eGFR <60 ml/min per 1.73 m^2^; models were adjusted for eGFR, eGFR slope, age, and all available comorbidities at baseline; continuous variables were incorporated via restricted cubic splines; *P* values denote interaction of sex and grouping variable. CVD, cardiovascular disease; eGFR, estimated glomerular filtration rate; HR, hazard ratio; KRT, kidney replacement therapy.

Figure 3 shows the results of the subgroup analyses. For the outcome of KRT, results consistent with our main findings were observed, with no interactions between sex and all our prespecified subgroups from Table 3. For the outcome of pre-KRT death, we also observed consistency with our main findings as the risk of death among men compared with women was always higher. However, multiplicative interactions suggested the effect size to be slightly higher in the presence (compared with absence) of diabetes or eGFR <60 ml/min per 1.73 m^2^ (*P* < 0.05 for both interactions).

### Secondary Outcome: All-Cause Death

**Figure 4:**
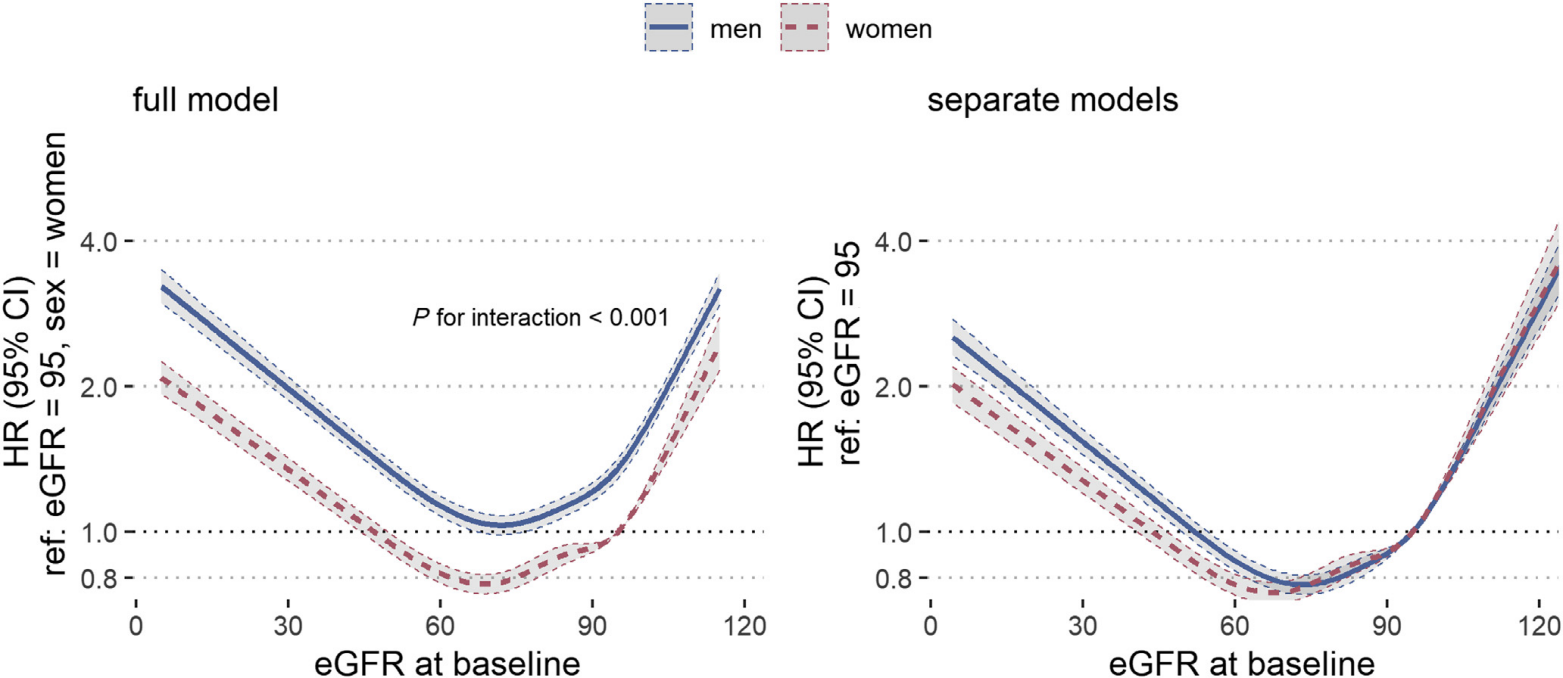
All-cause mortality HR by eGFR at baseline, per sex; adjusted for age, eGFR slope, and all available comorbidities at baseline (cardiovascular disease + diabetes + hypertension + 23 comorbidities, see Table 1 for full list); continuous variables were incorporated via restricted cubic splines; left: full model, including interaction for sex and eGFR; right: within-sex models. eGFR, estimated glomerular filtration rate; HR, hazard ratio; ref, reference.

**Figure 5:**
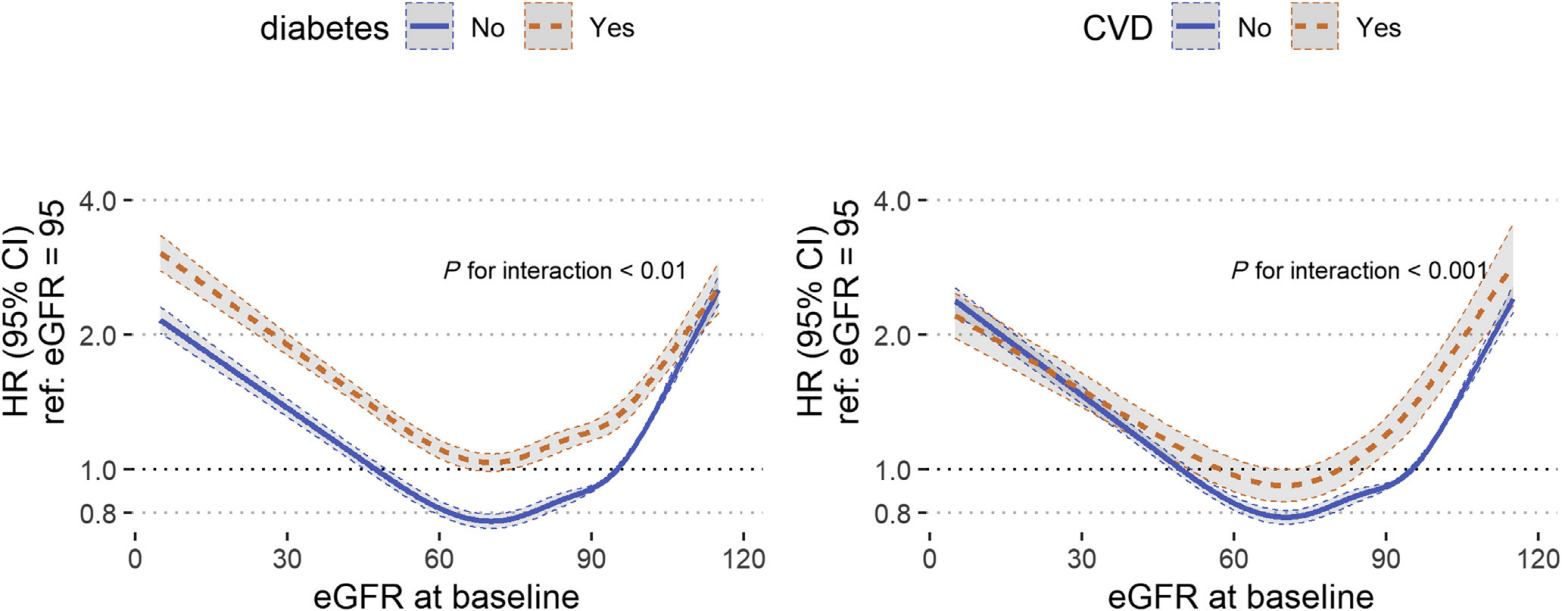
All-cause mortality HR by eGFR at baseline, per comorbidity; adjusted for age, eGFR slope, and all other available comorbidities at baseline (see Table 1 for full list); continuous variables were incorporated via restricted cubic splines; left: diabetes yes/no, with interaction of diabetes diagnosis and eGFR; right: CVD yes/no (i.e., congestive heart failure, peripheral vascular disorders or valvular disease) with interaction of CVD diagnosis and eGFR. CVD, cardiovascular disease; eGFR, estimated glomerular filtration rate; HR, hazard ratio; ref, reference.

Figure 4 depicts all-cause mortality hazard ratios with 95% CIs, by sex and with respect to baseline eGFR (reference eGFR = 95 ml/min per 1.73 m^2^). The left panel denotes the full model with women as reference group, with an annotation for the interaction *P* value between sex and eGFR (*P* < 0.001). The right panel compares the 2 within-sex models. In both sexes, we observed a J-shaped curve with strongly increasing mortality hazards in decreased baseline eGFR. The full model shows the overall higher mortality in men, compared with women (male-to-female HR for all-cause death: 1.35 [95% CI 1.33–1.38]). Effect modification analysis by diabetes and CVD diagnosis, respectively, is shown in Figure 5. In both models, we observed curves similar to the results by sex. While the interactions between diabetes and eGFR, and CVD and eGFR were both significant (eGFR × diabetes: *P* < 0.01, eGFR × CVD: *P* < 0.001), a diabetes diagnosis had a much stronger effect on eGFR-dependent mortality hazards than CVD.

### Sensitivity Analysis

All sensitivity analyses are summarized in Supplementary Table S2. In sensitivity analyses 1 and 2, we changed the predefined age cutoff of 45 years to ± 5 years (i.e., 40 and 50 years cutoff), so that additional, respectively fewer creatinine records were included. In sensitivity analysis 3, we excluded eGFR slope in the models, such that subjects could be included where slope estimation was infeasible (i.e., subjects recorded only once, or less than a year). For sensitivity analysis 4, we defined the timescale of the time-to-event analysis to the subject’s age. In sensitivity analysis 5, we dissected KRT into the competing events dialysis initiation and pre-emptive transplantation. Of the 364 KRT events in our study (compare Table 2), only 56 were pre-emptive transplantations (occurring in 36 men, 20 women), while the other 308 were dialysis initiation events (occurring in 182 men, 126 women). The results from all sensitivity analyses were consistent with the primary analysis.

## Discussion

In this study, we examined sex-specific differences in the competing events KRT initiation and pre-KRT death and all-cause mortality by kidney function in a noninstitutionalized general population cohort. Despite a higher CKD prevalence in women, we found that men in our study population had both a higher likelihood to receive KRT (male-to-female csHR 1.41 [95% CI 1.13–1.76]) and a higher chance to die without ever receiving KRT (male-to-female csHR 1.36 [95% CI 1.33–1.38]). All-cause mortality by kidney function indicated some sex differences but not in a manner that would contribute to observed sex disparities in CKD management.

Several hypotheses have been proposed to explain the discrepancy between a higher CKD prevalence among women but more men than women receiving KRT.^8^^,^^27^ These can broadly be summarized into biological factors on the one hand and nonbiological influences on the other. The first include the different comorbidity burdens between sexes,^4^ although this may be intermingled with factors such as physical activity, smoking, obesity, or other lifestyle factors that are not necessarily “biological variables.” Furthermore, hormones in women are suspected to have a protective effect on CKD progression,^10^^,^^28^ but findings on this are inconsistent; for example, they could not be confirmed in a recent analysis on a CKD cohort similar to ours.^29^ Arguably the most prominent hypothesis is the potentially faster progression of CKD in men. While there have been controversial findings about this hypothesis in the past, recent research collectively agrees that CKD progression is faster in men than in women.^15^^,^^16^^,^^29^

Nonbiological influences for KRT initiation include a suspected higher tendency for women to choose conservative treatment in comparison to men,^8^^,^^17^ and maybe more favorable economic and social circumstances of men,^4^ although the latter seems unlikely to be a strong influence in wealthy countries with universal health care, such as Sweden. Furthermore, in the United States, it has been shown that women in the general population are less aware of their CKD, both in early and late stages,^30^ that men are better prepared with arteriovenous fistulas at dialysis initiation,^31^ and that women on dialysis have lower chances to enter wait-lists for kidney transplantation.^32^ All of these reports indicate better CKD diagnosis and treatment for men.

Because of the reported sex differences in the management and identification of CKD, the ideal cohort to explore our research question of differing chances to receive KRT is one that represents the overall population, because sex bias may have affected who is included in nephrology-referred cohorts. Filling this knowledge gap, we observed in our cohort that women constituted the majority of subjects with G3 to G4 CKD, while more men had G5 CKD at study baseline. This finding is consistent with previous reports.^8^^,^^9^^,^^27^ The higher proportion of men with very low kidney function may partly be explained by a faster progression of CKD in men.^15^^,^^16^ The number of KRT initiations per 100,000 person years was nearly twice as high for men as for women (men: 26.3, women: 14.6 events per 100,000 person years), which was also reflected in an unadjusted male-to-female csHR for KRT of 1.795 (95% CI 1.456–2.214). To find evidence for nonbiological influences in sex-specific KRT initiation, we adjusted the hazards for all available biological variables, namely, a wide range of comorbidities as well as age and markers of kidney function. Still, men in our cohort had 41% (95% CI 13%–76%) higher chances than women to initiate KRT after adjusting for all these factors.

It has been proposed that in addition to a less rapid eGFR decline among women, higher death risks in the setting of CKD unawareness may also occur, such that women who might relatively more often be unaware of kidney disease could have a higher competing risk of mortality.^8^ We found no direct evidence for this hypothesis in our analysis, as men were also more likely than women to die before reaching KRT. One might argue that this assessment is incomplete, as men typically have higher all-cause mortality rates in time-to-event analyses. If all-cause mortality is higher in men, but more men than women initiate dialysis before dying, more deaths from men in comparison to women would be censored in the competing risks analysis; hence, the male-to-female csHR for pre-KRT death should be lower than the male-to-female HR for all-cause death. However, the fully adjusted male-to-female HR of all-cause death was 1.35 (95% CI 1.33–1.38) and thus is in line with the pre-KRT death csHR from the competing risks analysis. Furthermore, in the effect modification analysis summarized in Figure 3, we observed significantly increased risks of pre-KRT death for men in CKD risk factor subgroups, which is also contrary to what would be expected if women with CKD had an elevated risk of pre-KRT death. Hence, in our data, we found no evidence for a higher risk of pre-KRT death in women compared with men.

It has also been suspected that mortality with respect to kidney decline follows a steeper trajectory for women,^18^ which we could not confirm in this study. Sex-specific mortality by eGFR at baseline showed similar trends for men and women, albeit a statistically significant sex interaction (*P* < 0.001). However, as can best be seen in the within-sex models in the left panel of Figure 4, the largest differences in mortality by baseline eGFR were observed below 60 ml/min per 1.73 m^2^, where the HR (referenced to normal kidney function) was in fact higher in men.

Major strengths of the present study are the large sample size with complete coverage of the Stockholm region, which is crucial for a rare event such as KRT initiation, and the fact that the data were derived from a noninstitutionalized, general population cohort with a sufficiently long follow-up. Despite the high representativeness of the Stockholm population in SCREAM, it should be kept in mind that participants are included on the basis of health care use and creatinine testing, possibly inferring overrepresentation of certain subpopulations (i.e., older) and sicker patients. In addition, women were slightly overrepresented in SCREAM, and coverage of the 45 to 64 years age group was different between the sexes (80% for women, 76% for men). The main limitation of this study was the lack of lifestyle (e.g., smoking, obesity) and socioeconomic information or other nonbiological data. Because our aim was to find evidence for biological influences of sex-specific KRT initiation, we adjusted our analysis for available biological factors and interpreted the remaining sex discrepancies as an indication for nonbiological effects. A further potentially valuable exposure would have been premenopausal and postmenopausal status for women, to adjust for the suspected protective effect of hormones in women with respect to CKD progression. However, subgroup analyses did not show significant differences in KRT initiation for ages >60 years. In addition, the increase of the age cutoff from 45 to 50 years in the inclusion criteria for the creatinine measurements did not substantially alter the results. Another point of discussion may lie in the calculation of the eGFR slopes, which were intended to address the widespread theory that faster CKD progression is the main driver of sex disparities in CKD treatment. Our eGFR slope estimates were based on only 1 year of observation with potentially few creatinine measurements, which may have introduced bias. Furthermore, although creatinine measurements are common in routine outpatient care, it is probable that subjects with repeated creatinine measurements within a year were predominantly patients with comorbidities, on medication requiring monitoring, or had reduced kidney function. These more regularly monitored patients would in turn bias the eGFR slope estimation in the mixed effects model. This bias could explain why our mean eGFR slopes as reported in Table 1 are similar to eGFR slopes observed in CKD cohorts,^16^ although here, they describe a general population. Furthermore, for those subjects who developed CKD during follow-up, data on the primary cause of CKD was not available, such that differences between men and women, if any, could have contributed to differing KRT initiation rates. Importantly, we also acknowledge that we were unable to address whether gender-specific^33^ behavior (i.e., the existence of a “gender story” behind our data^34^) might further explain the associations we have identified between men versus women for KRT initiation. Finally, because of the design of observational study, there is always a risk of unmeasured confounding. Nevertheless, in view of at least 3 previous sex-specific analyses of mortality and KRT initiation from CKD cohorts of the United States,^16^ Sweden,^29^ and Italy,^35^ it was an important opportunity to fill the knowledge gap on the general population.

In summary, we found evidence that men were more likely to initiate KRT than women, which could not be explained by age, kidney function decline, and a wide range of comorbidities. This indication of sex disparities in kidney disease management should be further investigated, ideally in general population data including nonbiological information and with better assessment of kidney function decline. A broader understanding where and why women might be disadvantaged might eventually allow equalizing diagnosis and treatment of CKD among all parts of society.

## Disclosure

All the authors declared no competing interests.

## Supplementary Material

Supplementary File (PDF)

**Table S1.** ICD-10 codes used for the Elixhauser Comorbidity Score.

**Table S2.** Sensitivity Analyses.

**STROBE Statement** (PDF)
