# Supplementary material for: Kidney Function, Kidney Replacement Therapy, and Mortality in Men and Women
Source: Kidney Int Rep. 2021 Dec 27;7(3):444–54. doi: 10.1016/j.ekir.2021.12.024 (PMC8897304; doi:10.1016/j.ekir.2021.12.024)
Supplement: Supplementary File (PDF) [file mmc1.pdf]

Supplementary Table 1: ICD-10 codes used for the Elixhauser Comorbidity Score

**Comorbidity**	**ICD-10 Codes**
Congestive heart failure	I09.9, I11.0, I13.0, I13.2, I25.5, I42.0, I42.5 - I42.9, I43.x, I50.x, P29.0
Cardiac arrhythmias	I44.1 - I44.3, I45.6, I45.9, I47.x - I49.x, R00.0, R00.1, R00.8, T82.1, Z45.0, Z95.0
Valvular disease	A52.0, I05.x - I08.x, I09.1, I09.8, I34.x - I39.x, Q23.0 - Q23.3, Z95.2 - Z95.4
Pulmonary circulation disorders	I26.x, I27.x, I28.0, I28.8, I28.9
Peripheral vascular disorders	I70.x, I71.x, I73.1, I73.8, I73.9, I77.1, I79.0, I79.2, K55.1, K55.8, K55.9, Z95.8, Z95.9
Hypertension, uncomplicated	I10.x
Hypertension, complicated	I11.x - I13.x, I15.x
Paralysis	G04.1, G11.4, G80.1, G80.2, G81.x, G82.x, G83.0 - G83.4, G83.9
Other neurological disorders	G10.x - G13.x, G20.x - G22.x, G25.4, G25.5, G31.2, G31.8, G31.9, G32.x, G35.x - G37.x, G40.x, G41.x, G93.1, G93.4, R47.0, R56.x
Chronic pulmonary disease	I27.8, I27.9, J40.x - J47.x, J60.x - J67.x, J68.4, J70.1, J70.3
Diabetes, uncomplicated	E10.0, E10.1, E10.9, E11.0, E11.1, E11.9, E12.0, E12.1, E12.9, E13.0, E13.1, E13.9, E14.0, E14.1, E14.9
Diabetes, complicated	E10.2 - E10.8, E11.2 - E11.8, E12.2 - E12.8, E13.2 - E13.8, E14.2 - E14.8
Hypothyroidism	E00.x - E03.x, E89.0
Liver disease	B18.x, I85.x, I86.4, I98.2, K70.x, K71.1, K71.3 - K71.5, K71.7, K72.x - K74.x, K76.0, K76.2 - K76.9, Z94.4
Peptic ulcer disease, excluding bleeding	K25.7, K25.9, K26.7, K26.9, K27.7, K27.9, K28.7, K28.9
AIDS/HIV	B20.x - B22.x, B24.x
Lymphoma	C81.x - C85.x, C88.x, C96.x, C90.0, C90.2
Metastatic cancer	C77.x - C80.x
Solid tumour without metastasis	C00.x - C26.x, C30.x - C34.x, C37.x - C41.x, C43.x, C45.x - C58.x, C60.x - C76.x, C97.x
Rheumatoid arthritis/collagen vascular diseases	L94.0, L94.1, L94.3, M05.x, M06.x, M08.x, M12.0, M12.3, M30.x, M31.0 - M31.3, M32.x - M35.x, M45.x, M46.1, M46.8, M46.9
Coagulopathy	D65 - D68.x, D69.1, D69.3 - D69.6
Obesity	E66.x
Weight loss	E40.x - E46.x, R63.4, R64
Fluid and electrolyte disorders	E22.2, E86.x, E87.x
Blood loss anaemia	D50.0
Deficiency anaemia	D50.8, D50.9, D51.x - D53.x
Alcohol abuse	F10, E52, G62.1, I42.6, K29.2, K70.0, K70.3, K70.9, T51.x, Z50.2, Z71.4, Z72.1
Drug abuse	F11.x - F16.x, F18.x, F19.x, Z71.5, Z72.2
Psychoses	F20.x, F22.x - F25.x, F28.x, F29.x, F30.2, F31.2, F31.5
Depression	F20.4, F31.3 - F31.5, F32.x, F33.x, F34.1, F41.2, F43.2

Supplementary Table 2: Sensitivity Analyses of main results. Male-to-female cause specific hazard ratios (csHR) with 95% confidence intervals of the competing events kidney replacement therapy (KRT) and pre-KRT deaths, as well as all-cause death hazard ratios (HR), in different sensitivity analyses scenarios; cardiovascular disease (CVD) includes heart failure, peripheral vascular disorders or valvular disease; fully adjusted models include diabetes, hypertension, the three CVD factors and all other 23 comorbidities depicted in **Table 1.**

Sensitivity Analysis 1		adjustments	KRT m-to-f csHR	pre-KRT death m-to-f csHR	all-cause death m-to-f HR
use of all records were subjects were older than 40 years of age (instead of 45 year in main analysis)	Model 1	unadjusted	1.816 (1.484,2.224)	1.039 (1.022,1.056)	1.039 (1.023,1.056)
	Model 2	age	1.771 (1.446,2.170)	1.460 (1.437,1.485)	1.460 (1.436,1.484)
	Model 3	age + diabetes	1.577 (1.285,1.935)	1.434 (1.410,1.458)	1.433 (1.410,1.457)
	Model 4	age + diabetes + hypertension	1.592 (1.297,1.954)	1.433 (1.409,1.457)	1.432 (1.409,1.456)
	Model 5	age + diabetes + hypertension + CVD	1.557 (1.268,1.912)	1.395 (1.372,1.418)	1.395 (1.372,1.418)
	Model 6	age + diabetes + hypertension + CVD + eGFR + eGFR slope	1.424 (1.152,1.761)	1.392 (1.369,1.415)	1.391 (1.368,1.414)
	Model 7	age + diabetes + hypertension + CVD + eGFR + eGFR slope + 23 comorbidities	1.441 (1.154,1.801)	1.356 (1.332,1.379)	1.355 (1.332,1.378)

Sensitivity Analysis 2		adjustments	KRT m-to-f csHR	pre-KRT death m-to-f csHR	all-cause death m-to-f HR
use of all records were subjects were older than 50 years of age (instead of 45 year in main analysis)	Model 1	unadjusted	1.754 (1.412,2.179)	1.042 (1.025,1.059)	1.042 (1.025,1.059)
	Model 2	age	1.694 (1.362,2.106)	1.463 (1.438,1.487)	1.462 (1.438,1.487)
	Model 3	age + diabetes	1.511 (1.213,1.882)	1.436 (1.412,1.460)	1.436 (1.412,1.460)
	Model 4	age + diabetes + hypertension	1.544 (1.240,1.924)	1.435 (1.411,1.459)	1.434 (1.411,1.459)
	Model 5	age + diabetes + hypertension + CVD	1.500 (1.204,1.870)	1.397 (1.373,1.420)	1.396 (1.373,1.420)
	Model 6	age + diabetes + hypertension + CVD + eGFR + eGFR slope	1.333 (1.069,1.663)	1.389 (1.366,1.413)	1.388 (1.365,1.412)
	Model 7	age + diabetes + hypertension + CVD + eGFR + eGFR slope + 23 comorbidities	1.342 (1.063,1.694)	1.355 (1.332,1.379)	1.354 (1.331,1.378)

Sensitivity Analysis 3		adjustments	KRT m-to-f csHR	pre-KRT death m-to-f csHR	all-cause death m-to-f HR
without the use of eGFR slope in Model 7, includes also subjects with only one measurement or which were observed less than a year	Model 1	unadjusted	1.847 (1.545,2.210)	1.018 (1.004,1.032)	1.019 (1.005,1.033)
	Model 2	age	1.836 (1.534,2.198)	1.471 (1.451,1.492)	1.472 (1.451,1.492)
	Model 3	age + diabetes	1.633 (1.363,1.957)	1.447 (1.427,1.467)	1.447 (1.427,1.467)
	Model 4	age + diabetes + hypertension	1.666 (1.390,1.997)	1.449 (1.429,1.470)	1.450 (1.430,1.470)
	Model 5	age + diabetes + hypertension + CVD	1.639 (1.367,1.965)	1.418 (1.399,1.438)	1.419 (1.399,1.439)
	Model 6	age + diabetes + hypertension + CVD + eGFR	1.336 (1.112,1.604)	1.408 (1.388,1.427)	1.408 (1.388,1.427)
	Model 7	age + diabetes + hypertension + CVD + eGFR + 23 comorbidities	1.383 (1.143,1.674)	1.369 (1.350,1.389)	1.370 (1.350,1.389)

Sensitivity Analysis 4		adjustments	KRT m-to-f csHR	pre-KRT death m-to-f csHR	all-cause death m-to-f HR
subject age as timescale for the time-to-event data; cox model which allowed for flexible entrance times into the study	Model 1	unadjusted	1.724 (1.397,2.127)	1.461 (1.437,1.486)	1.461 (1.437,1.485)
	Model 2	diabetes	1.541 (1.247,1.905)	1.435 (1.411,1.459)	1.434 (1.410,1.458)
	Model 3	diabetes + hypertension	1.568 (1.268,1.938)	1.433 (1.410,1.457)	1.433 (1.409,1.457)
	Model 4	diabetes + hypertension + CVD	1.528 (1.235,1.890)	1.396 (1.373,1.419)	1.395 (1.372,1.419)
	Model 5	age + CVD + diabetes + eGFR + eGFR slope	1.355 (1.095,1.678)	1.392 (1.369,1.415)	1.391 (1.368,1.414)
	Model 6	age + CVD + diabetes + eGFR + eGFR slope + 23 comorbidities	1.392 (1.112,1.741)	1.357 (1.334,1.381)	1.356 (1.333,1.380)

Sensitivity Analysis 5			DIALYSIS m-to-f csHR	KTX m-to-f csHR	pre-KRT death m-to-f csHR	all-cause death m-to-f HR
KTX and DIALYSIS as separated competing events	Model 1	unadjusted	1.736 (1.383,2.178)	2.170 (1.256,3.749)	1.039 (1.022,1.056)	1.039 (1.023,1.056)
	Model 2	age	1.709 (1.361,2.147)	1.945 (1.126,3.361)	1.462 (1.438,1.487)	1.462 (1.438,1.486)
	Model 3	age + diabetes	1.506 (1.197,1.895)	1.883 (1.087,3.264)	1.436 (1.412,1.460)	1.435 (1.412,1.459)
	Model 4	age + diabetes + hypertension	1.538 (1.222,1.936)	1.878 (1.084,3.254)	1.434 (1.411,1.458)	1.434 (1.410,1.458)
	Model 5	age + diabetes + hypertension + CVD	1.488 (1.181,1.874)	1.913 (1.104,3.316)	1.396 (1.373,1.420)	1.396 (1.373,1.419)
	Model 6	age + diabetes + hypertension + CVD + eGFR + eGFR slope	1.317 (1.045,1.660)	1.717 (0.991,2.977)	1.391 (1.368,1.414)	1.390 (1.367,1.413)
	Model 7	age + diabetes + hypertension + CVD + eGFR + eGFR slope + 23 comorbidities	1.390 (1.089,1.775)	NA (singular model matrix)	1.355 (1.332,1.379)	1.354 (1.331,1.378)

Modified STROBE Statement—checklist of items that should be included in reports of observational studies (Cohort/Cross-sectional and case-control studies)

	Item No	Recommendation	Comment from the authors
**Title and abstract**	1	(*a*) Indicate the study's design with a commonly used term in the title or the abstract	The study design is mentioned in the title as well as the abstract
		(*b*) Provide in the abstract an informative and balanced summary of what was done and what was found	see Abstract, page 2
**Introduction**			
Background/rationale	2	Explain the scientific background and rationale for the investigation being reported	The scientific background and the study rationale are explained in the introduction, pages 4 and 5
Objectives	3	State specific objectives, including any prespecified hypotheses	The study objective is explained in the introduction, page 5
**Methods**			
Study design	4	Present key elements of study design early in the paper	The methods section starts with a detailed description of the study design, page 5
Setting	5	Describe the setting, locations, and relevant dates, including periods of recruitment, exposure, follow-up, and data collection	The study population is described in the methods section, pages 5 and 6
Participants	6	(*a*) *Cohort study*—Give the eligibility criteria, and the sources and methods of selection of participants. Describe methods of follow-up<br><br>*Case-control study*—Give the eligibility criteria, and the sources and methods of case ascertainment and control selection. Give the rationale for the choice of cases and controls<br><br>*Cross-sectional study*—Give the eligibility criteria, and the sources and methods of selection of participants	Inclusion and exclusion criteria are described on page 6
Variables	7	Clearly define all outcomes, exposures, predictors, potential confounders, and effect modifiers. Give diagnostic criteria, if applicable	All exposures and outcomes are described in detail on pages 6 and 7
Data sources/ measurement	8*	For each variable of interest, give sources of data and details of methods of assessment (measurement).	Sources of all variables are explained within the study design and study cohort descriptions, pages 5-7
Bias	9	Describe any efforts to address potential sources of bias	Biases are addressed by careful definition of the study cohort (page 5) as well as adjustments

			of the statistical models (page 7-9)
Study size	10	Explain how the study size was arrived at (if applicable)	-
Quantitative variables	11	Explain how quantitative variables were handled in the analyses. If applicable, describe which groupings were chosen and why	-
Statistical methods	12	(*a*) Describe all statistical methods, including those used to control for confounding	All methods used are described in detail, page 8
		(*b*) Describe any methods used to examine subgroups and interactions	Subgroup and effect modification analysis are described in the statistical methods, pages 8 and 9
		(*c*) Explain how missing data were addressed	We only encountered missing data in the initial data cleaning process, shown on the study flowchart, Figure 1
		(*d*) *Cohort study*—If applicable, explain how loss to follow-up was addressed<br>*Case-control study*—If applicable, explain how matching of cases and controls was addressed<br>*Cross-sectional study*—If applicable, describe analytical methods taking account of sampling strategy	We had complete information on the outcome variables, hence we didn't encounter loss to follow-up
		(*e*) Describe any sensitivity analyses	We implemented subgroup analysis of the primary and secondary objective, which also served as sensitivity analysis, see pages 8 and 9
**Results**			
Participants	13*	(a) Report numbers of individuals at each stage of study—eg numbers potentially eligible, examined for eligibility, confirmed eligible, included in the study, completing follow-up, and analyzed	Detailed numbers of subjects after every exclusion or inclusion criterion were reported in Figure 1
		(c) **Use of a flow diagram**	See Figure 1
Descriptive data	14*	(a) Give characteristics of study participants (eg demographic, clinical, social) and information on exposures and potential confounders	The study cohort was summarized in detail in Table 1
		(b) Indicate number of participants with missing data for each variable of interest	-

		(c) *Cohort study*—Summarise follow-up time (eg, average and total amount)	Follow-up times were reported in Table 2
Outcome data	15*	*Cohort study*—Report numbers of outcome events or summary measures over time	Outcome events were reported in Table 2
		Case-control study—Report numbers in each exposure category, or summary measures of exposure	-
		Cross-sectional study—Report numbers of outcome events or summary measures	-
Main results	16	(*a*) Give unadjusted estimates and, if applicable, confounder-adjusted estimates and their precision (eg, 95% confidence interval). Make clear which confounders were adjusted for and why they were included	Unadjusted and adjusted estimates were reported in Table 3
Other analyses	17	Report other analyses done—eg analyses of subgroups and interactions, and sensitivity analyses	Subgroup analysis were shown in Figure 3 and Figure 4
**Discussion**			
Key results	18	Summarise key results with reference to study objectives	The key results were described at the beginning of the discussion, page 11
Limitations	19	Discuss limitations of the study, taking into account sources of potential bias or imprecision. Discuss both direction and magnitude of any potential bias	The limitations of our study are discussed on page 14
Interpretation	20	Give a cautious overall interpretation of results considering objectives, limitations, multiplicity of analyses, results from similar studies, and other relevant evidence	Interpretation of the results, implications for the objectives, comparisons and evidence to results from other studies, and the limitations were outlined in the discussion, pages 12-15
Generalisability	21	Discuss the generalisability (external validity) of the study results	The large sample size and the discussed measures to control for confounding support the generalizability of the study.

*Give information separately for cases and controls in case-control studies and, if applicable, for exposed and unexposed groups in cohort and cross-sectional studies.

Note: An Explanation and Elaboration article discusses each checklist item and gives methodological background and published examples of transparent reporting. The STROBE checklist is best used in conjunction with this article (freely available on the Web sites of PLoS Medicine at http://www.plosmedicine.org/, Annals of Internal Medicine at http://www.annals.org/, and Epidemiology at http://www.epidem.com/). Information on the STROBE Initiative is available at www.strobe-statement.org.
